# Automated and unsupervised detection of malarial parasites in microscopic images

**DOI:** 10.1186/1475-2875-10-364

**Published:** 2011-12-13

**Authors:** Yashasvi Purwar, Sirish L Shah, Gwen Clarke, Areej Almugairi, Atis Muehlenbachs

**Affiliations:** 1 Department of Chemical and Materials Engineering, University of Alberta, Edmonton, Canada; 2 Alberta Health Services, Province of Alberta, Edmonton, Canada; 3 Departments of Pathology and Laboratory Medicine, University of Washington, Seattle, USA

## Abstract

**Background:**

Malaria is a serious infectious disease. According to the World Health Organization, it is responsible for nearly one million deaths each year. There are various techniques to diagnose malaria of which manual microscopy is considered to be the gold standard. However due to the number of steps required in manual assessment, this diagnostic method is time consuming (leading to late diagnosis) and prone to human error (leading to erroneous diagnosis), even in experienced hands. The focus of this study is to develop a robust, unsupervised and sensitive malaria screening technique with low material cost and one that has an advantage over other techniques in that it minimizes human reliance and is, therefore, more consistent in applying diagnostic criteria.

**Method:**

A method based on digital image processing of Giemsa-stained thin smear image is developed to facilitate the diagnostic process. The diagnosis procedure is divided into two parts; enumeration and identification. The image-based method presented here is designed to automate the process of enumeration and identification; with the main advantage being its ability to carry out the diagnosis in an unsupervised manner and yet have high sensitivity and thus reducing cases of false negatives.

**Results:**

The image based method is tested over more than 500 images from two independent laboratories. The aim is to distinguish between positive and negative cases of malaria using thin smear blood slide images. Due to the unsupervised nature of method it requires minimal human intervention thus speeding up the whole process of diagnosis. Overall sensitivity to capture cases of malaria is 100% and specificity ranges from 50-88% for all species of malaria parasites.

**Conclusion:**

Image based screening method will speed up the whole process of diagnosis and is more advantageous over laboratory procedures that are prone to errors and where pathological expertise is minimal. Further this method provides a consistent and robust way of generating the parasite clearance curves.

## Background

Malaria is an infectious disease and causes serious health problems; half of the world's population, particularly in the developing countries is at risk of malaria [1]. According to the World Health Organization (WHO), malaria causes approximately nearly million deaths and over 250 million infections every year. and is caused by parasites of the genus *Plasmodium*, of which *Plasmodium falciparum *contributes 98% of deaths [1]. The diagnosis of the infections due to *P. falciparum *is still carried out via manual procedures especially in developing countries. Although there are advanced methods of diagnosis [2], manual microscopy of blood films on slides is still considered to be the gold standard. Manual microscopy has advantage over other techniques in that it is both sensitive and specific [3]. One of the disadvantages of diagnosis using manual microscopy methods is that it requires extensive human intervention during the diagnostic process which can often lead to late and sometimes erroneous diagnosis. The microscopist requires extensive training to gain expertise in the diagnosis, and because of the sheer volume of the samples that need to be analysed, the method is not consistent and is dependent upon blood smear and stain quality, microscope quality and the expertise of the microscopist.

Some of the problems of manual microscopy can be overcome by exploring computer based, specifically image-based, diagnostic methods. The aim of this study is to outline a semi-automatic diagnosis method based on image processing and one that provides a reliable and consistent solution. The literature contains descriptions and details of several computer vision or image-based algorithms [4-9]. However, most of these algorithms are supervised and complex, that is they need manual intervention or calibration. Considering the high fatality rate and huge volumes of samples that need to be analysed we need a sensitive, practical and robust method with minimum human intervention. In this context, computer based diagnosis can help in the rapid, accurate and consistent identification of true malaria cases, ensuring that only those patients with malaria are treated.

Manual microscopy is carried out by examining thin blood films on slides under the microscope and reporting the percentage of parasitaemia (i.e. number of infected red blood cells (iRBCs) for over 100 microscopic fields). Microscopists also need to identify parasite morphology by various life cycle stages for speciation, described in The WHO practical microscopy guide [3]. Giemsa staining is most widely used to highlight the parasites. The disadvantage of Giemsa is that it also stains other blood film features, such as white blood cells, platelets, and slide artefacts, such as dust particles. This problem of other stained objects needs to be considered carefully when comparing results of automated image-based diagnosis with manual microscopy. It is proposed that the method presented here be mainly used as a consistent screening tool to identify patients who are likely to have parasitaemia. This would significantly reduce load on medical technologists who can then focus their attention on those patients with positive tests to confirm presence of parasites and the level of parasitaemia with manual slide examination.

Each image as discussed below may not represent one complete microscopic field. Instead each image is considered as a segment of a field. The focus of this study is on the application of image analysis on low resolution images, which directly implies that smaller segments can be drawn from a larger field and analyzed separately for accurate results. This approach mimics the procedure that a pathologist would carry out as most pathologists or microbiologists would examine a section of a larger field to obtain accurate result.

In general thick smear screening is more sensitive than thin smear to detect parasites with a human microscopist. In endemic areas the thick smear is examined first and the thin smear secondarily used for speciation and to assess parasitemia. In general due to time constraints and visual human fatigue only 100-300 high-power fields (hpf) are examined on a thin smear- that is only a small fraction of the fields present on a traditional glass slide are actually examined.

The method proposed in this manuscript presents an unsupervised screening method for thin smear analysis, which has potential to be integrated into a diagnostic platform. For the proposed method to have clinical utility, close to 1,000 hpf would need to be analysed- this could happen on a traditional glass slide or on a yet-to-be-developed substrate. The image capture technology has yet to be developed suitable for a tropical laboratory. A 10-20 parasites/μL would be feasible on a thin smear generated with 10 μL of blood and > 1,000 hpf analysed.

## Methods

An automated diagnostic method can be developed by understanding the diagnostic process and representing it by a specifically tailored image processing based algorithm. The image processing based algorithm should perform diagnosis more or less imitating the manual microscopy. The algorithm should be capable of operating in an unsupervised environment and needs to be robust with minimal false negatives (leading to high sensitivity). The unsupervised nature of the proposed procedure should reduce human intervention, and in so doing speed up the diagnosis process. The algorithm should also be sensitive enough to capture parasites at all stages particular at the early stages of their life cycle and do this without missing any parasites irrespective of image variations. In order to perform diagnosis, the method must be capable of differentiating between parasite and artefacts. The majority of the image based diagnosis methods [4-9] reported in literature do not address this requirement.

**Figure 1:**
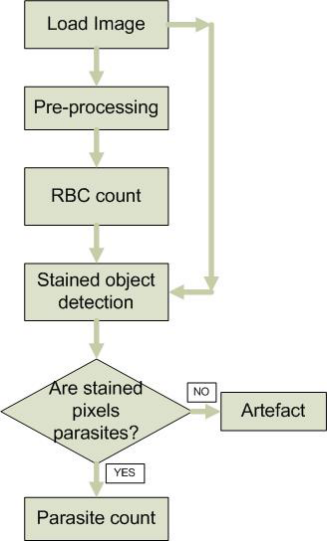
**Flowchart describing the steps of the proposed algorithm**.

The challenge to achieve this high degree of sensitivity to parasites with an ability to exclude artefacts and debris in an unsupervised environment was carried out by developing a very novel and simple statistical method for image classification. The image classification problem performs the following steps: i) RBC enumeration, ii) potential parasite identification and iii) reports parasitaemia by counting the number of iRBC per every 1000 RBCs considering each image corresponds to a section of microscopic field. The steps that constitute the image processing and image segmentation tasks are summarized in the flowchart shown in Figure 1. The image based diagnostic method described below was developed using MATLAB2007b computational platform.

### Materials

Anonymized thin blood film images were obtained from Alberta Health Services laboratories at the University of Alberta. The samples obtained mostly had low number of parasites in early stages (rings) of their life cycle. Such features are often hard to detect. In addition several samples did not have any parasites (negative controls). The samples were stained using a fast Giemsa protocol to highlight the parasites and were initially examined by haematopathologists with expertise in malaria diagnosis. Slide images were acquired using a charge coupled device (CCD) camera with different range of magnification. Some images had variable stain characteristics making computer-based detection more challenging. In total, 10 positive subject samples were examined and parasitaemia was reported as number of iRBCs per thousand RBCs. Thus, for each subject 10 images, each representing a section of microscopic field at 1,000 × magnification and containing approximately 100 RBCs were acquired. Ten (10) negative control cases were included in a blinded fashion to assess the specificity of the image-based method. In-total 20 cases were analysed i.e. 10 positive cases and 10 negative cases obtained from Alberta Health Services laboratories.

### Pre-processing

**Figure 2:**
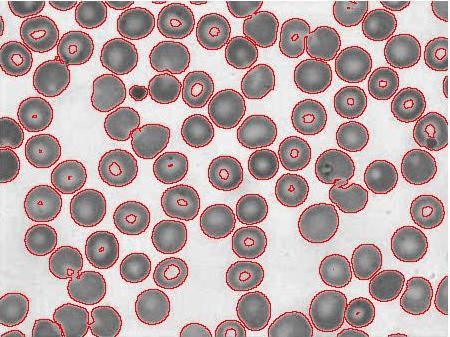
**Boundary extracted image using the Chan-Vese segmentation method**.

The purpose of pre-processing is to remove unwanted objects and noise from the image to facilitate image segmentation into meaningful regions. The steps required to carry out image pre-processing were implemented on low resolution images are as follows:

i) Load coloured (RGB) or gray scale image, the coloured image is converted to gray scale image. The contrast of the gray scale image is enhanced using local histogram equalization [10] to enhance the visibility of the parasites and RBC.

ii) The next and important step in image segmentation is to extract meaningful regions, or in other words, distinguish objects from background. The common way described in the literature is to use edge detection algorithms [10,11] but edge detection algorithms use gradient information followed by morphological boundry closing. Its not always easy to detect edges and location of object in presence of substantial noise. This makes edge detection algorithms computationally exhaustive and less sensitive. A method is required which can overcome the problems of edge detection algorithms. Chan-Vese *et al *[12,13] proposed energy minimization of the image to detect edges of objects embedded within an image. The goal of implementing Chan-Vese based boundary detection algorithm to segment image into meaningful regions, in our case separate RBC and artefacts from the background is shown in Figure 2.

**Figure 3:**
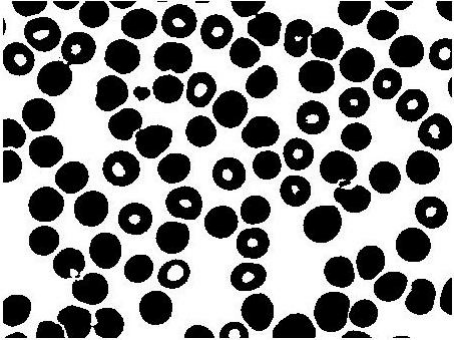
**Binary image from the Chan-Vese segmentation method**.

**Figure 4:**
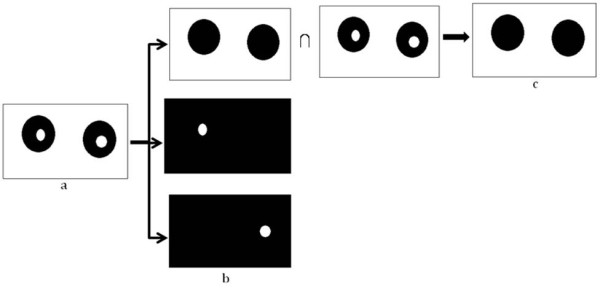
**Example to illustrate the 'hole-filling' algorithm: a) Dummy image with holes b) All the connected components c) Holes filled image after intersection**.

**Figure 5:**
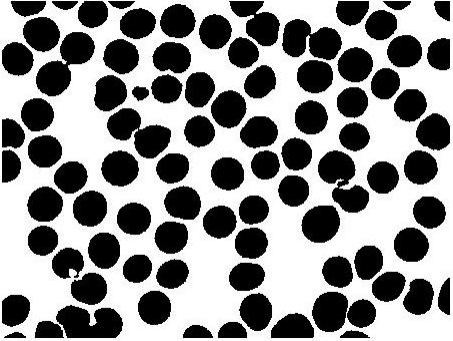
**Binary image after removing spurious boundaries**.

The binary image of statistically similar region generated after Chan-Vese segmentation distinguishing RBC and background, but because of biconcave shape of the RBC, the central pallor is assigned the same features as the background as shown in Figure 3. It is required to remove the central pallor in the RBC and to perform this task a 'hole filling' algorithm was designed as described with a simple illustrative example in Figure 4. The technique to fill the holes in the binary digital image is to extract the largest connected component among all the connected components. Connected component analysis extracts the information on pixel connectivity in a two-dimensional image by labelling connected pixels possessing same intensities. All the connected components were extracted as shown in Figure 4b and intersection of the largest connected component i.e. the background was performed with the original image shown in Figure 4c. The reason for explicitly designing a hole-filling method is to be able to obtain an average diameter of the RBCs. To do this we need to obtain a count of the RBCs. The central pallor could easily get construed as an RBC and, therefore, it had to be masked or the hole had to be filled to capture the total number of RBCs and their size distribution. Secondly, the hole-filling algorithm provides an extra piece of information about the size distribution of central pallor which can be useful in detecting other blood diseases.

iii) The hole-filled image is followed by minute erosion [10,11] using disk-shaped structuring element of radius 5 pixels, the resulting image after hole-filling and erosion is shown in Figure 5. The erosion will remove any isolated pixel(s) in the image thus reducing the number of artefacts.

### RBC count

The number of RBCs in the pre-processed binary image can be calculated if the total area occupied by RBCs is divided by the area of one RBC and approximating the total count to the nearest integer for the total number of RBCs. The challenge was to determine the accurate area of one RBC for each image without any prior information (because the size of RBCs depend on factors such as a patient's age, magnification etc). A first attempt to automate this procedure in an unsupervised algorithm was to obtain a size distribution of RBCs in pixel units. To accomplish this, it is observed that RBCs possess almost circular shape, this feature of RBC can be exploited and various radius circles can be fitted to the RBC to generate a circle size distribution. The mean area generated from circle size distribution was used to determine the total number of RBCs in the image. The problem of fitting radii of various sizes to RBCs was implemented using the popular 'Hough transform' [10,11].

Hough transform works as a powerful tool for image segmentation to extract pre-defined (line, circular, elliptical etc.) shapes in an image. Hough transform tries to determine if the group of pixels lie on a curve of specific shape. A commonly used version of Hough transform is used for extracting straight lines in the image. The method developed by Hough to extract straight lines can be extended to determine circles within an image. The circular Hough transform method is a modified from of conventional Hough transform, where the parameter equation for straight line is changed to the general equation of circle expressed in equation [1].

(1) $$\begin{array}{c} a=x-r\cos\phi \\ b=y-r\sin\phi \end{array}$$

Now given a gradient angle (ø) at an edge point (x, y), a and b can be computed for pre-defined radius (r) or for given radius range. The edge points lying on the same circle are assigned to one accumulator array and this process is repeated for each point on the image. The algorithm terminates when all edge points have been assigned to one of the accumulator arrays thus each accumulator array possess the centre and corresponding radius of the circle.

**Figure 6:**
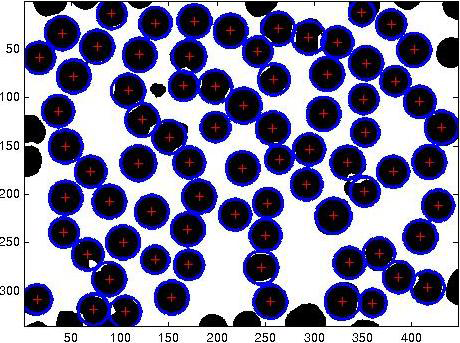
**Circles of different radii are detected on binary image after removing spurious boundaries**.

**Figure 7:**
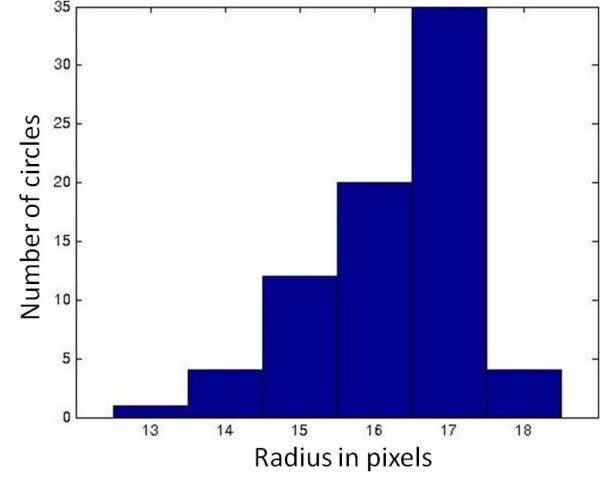
**Histogram of circle size distribution**.

**Figure 8:**
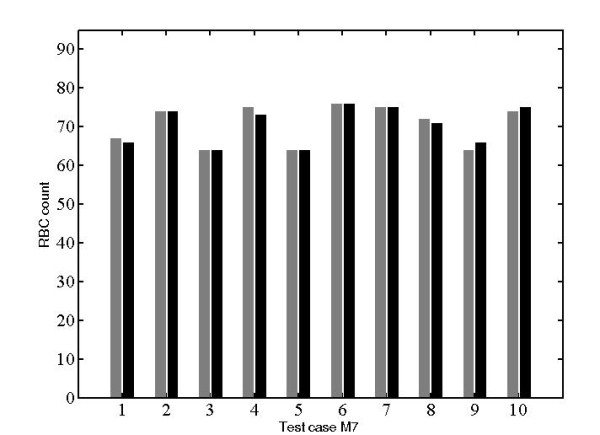
**RBC size histogram of subject M7**.

As can be observed from a typical slide shown in Figure 5, the RBCs are not all perfectly circular, which leads to the suggestion of using the Hough transform for ellipse fitting [14]. However literature studies [10,11] indicate that as the number of parameters increases, as it would in ellipse fitting, the algorithm becomes slower and furthermore it may have severe memory allocation problems. Therefore, the Hough transform for circle of different radii was implemented and the results demonstrate the efficacy of the method in that it allowed the feature generation to be very robust and completely unsupervised. An example of this implementation is shown in Figure 6 followed by circle size distribution shown in Figure 7. Table 1 and Figure 8 represents results obtained from the analysis of one randomly picked subject M7.

**Table 1:** Comparison of RBC count using the automated algorithm and its comparison with the manual count (edge cells at image boundaries were considered)

M7	Algorithm count	Manual count	ErroR
M7-1	67	66	1

M7-2	74	74	0

M7-3	64	64	0

M7-4	75	73	2*

M7-5	64	64	0

M7-6	76	76	0

M7-7	75	75	0

M7-8	72	71	1

M7-9	64	66	2*

M7-10	74	75	1

### Parasite detection

The next part of the proposed methodology is the detection of potential parasites in Giemsa-stained thin smear blood slide images. Considering that parasites are small stained objects embedded within the RBC, they are often distinguishable from the RBC plus other background 'image noise' and artefacts (artefacts represents platelets, WBC, dirt, dye crystals etc.) in the image. Although the literature is archived with several methods for image segmentation based on edge detection [9] or statistical methods using classifiers [6,7] for the detection of parasites, the edge detection method as discussed earlier is dependent on gradient information of the image and tends to generate undesired results if the parasites are barely visible or in early stages of their life cycle. On the other hand, classification methods tend to overcome these problems, but development of a classifier requires access to historical information making it a supervised learning method.

The primary objective in this study was to have a robust unsupervised method for the detection and enumeration of malaria parasites. In this context, the property of parasites being stained can be exploited to separate them from the RBC and the background. To accomplish this objective, an unsupervised, sensitive and reliable segmentation method, which extracts parasite information from the image, is proposed.

**Figure 9:**
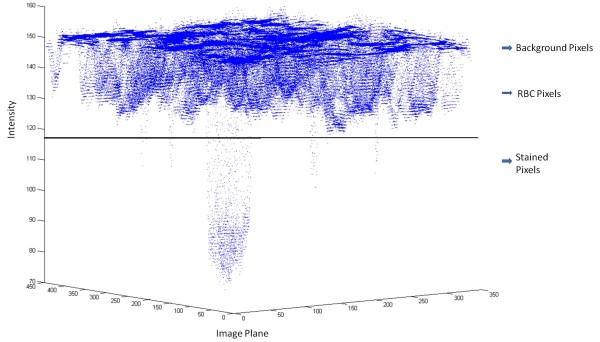
**Pixel intensity plot with random threshold**.

The visual inspection of pixel intensity data plot as shown in Figure 9 reveals the distinguishable clusters of background, RBC and stained pixels. The pixel intensity data plot suggests the use of a non-hierarchical method of clustering technique such as k-means clustering (KMC) [15] to segment the pixels into corresponding distinguishable groups. The advantage of clustering methods is to uniquely classify data, which in this case is based on the digital image, using an unsupervised learning methodology. These methods are also robust and can be easily implemented to achieve a desired level of image segmentation. The implementation of robust clustering method requires prior information of number of clusters and good initial guess of cluster centroids. Commercially available software packages (e.g. MATLAB) provide KMC as a tool for data clustering but these algorithms are not designed for image segmentation and in any case they require a good initial guess for the number of clusters and cluster centroids to have meaningful clustering. Figure 9 shows that the cluster of stained pixels, presumably representing parasites, has very few data points compared to RBCs and the background cluster. Clearly these giant data clusters easily overwhelm the small clusters, with the result that a clear demarcation between the RBCs and the parasites is not possible using conventional KMC.

**Figure 10:**
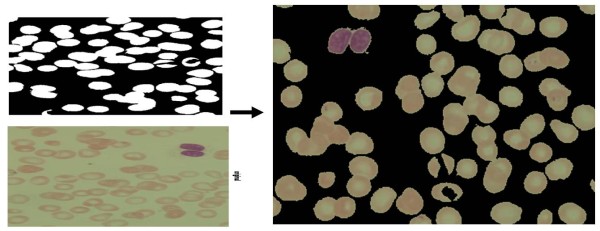
**Segmented image with background set to 'zero' pixel values**.

**Figure 11:**
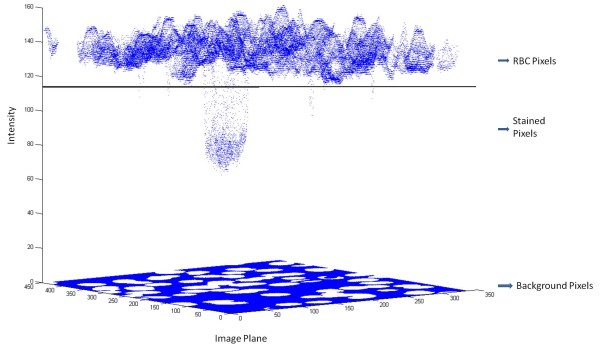
**Pixel intensity plot with 'background pixel intensity' values set to zero**.

To overcome the problems of unknown number of clusters, good initial guesses and retaining the information about small clusters in the presence of giant clusters, a modified KMC based algorithm has been developed. The problem of unknown number of clusters can be addressed as catching almost empty clusters, if KMC is initialized with more number of clusters than expected. If the algorithm encounters an empty cluster it reduces the number of cluster by one in an iterative process until the process reaches an optimum number of separable clusters. The problem of good initial guess was combined with the initialization of the clustering algorithm in order to facilitate the clustering process by segmenting the background cluster. The background cluster can be separated from the RBC and artefacts by superimposing the hole-filled image over the original RGB image as shown in Figure 10. The visual inspection of pixel intensity plot as shown in Figure 11 reveals that background intensity suppressed to zero makes the clusters more distinguishable. The pixel intensity of the background set to zero gives us the freedom to choose the initial centroids of equal weight between zero and maximum pixel intensity value. The sparseness of the pixel map combined with a good initial guess makes the KMC algorithm fast and robust. This initial guess serves as a default guess for all cases and thus rendering this as an unsupervised parasite detection and enumeration algorithm.

**Figure 12:**
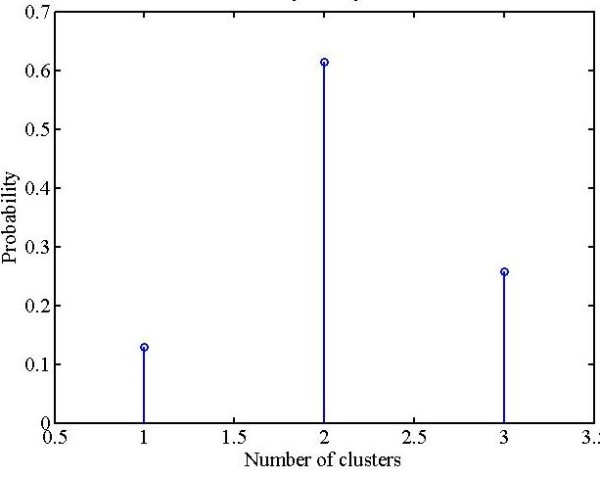
**Probability density function for 3 clusters**.

The parasite cluster as observed in Figures 9 and 11 is a weak cluster or in other words fewer pixels are stained. The small clusters (stained pixels) buried under a large population are clearly overwhelmed. Therefore, the small cluster information is lost during the process of clustering and hardly any of the small clusters appear as separate cluster(s). The solution to this problem is obtained by imparting higher weights to the small cluster(s) compared to the larger clusters. In this way the small cluster information is retained by making small cluster comparable to big clusters. The new modified form of KMC as designed with the modifications discussed above is defined as *probabilistic k-means clustering *(PKMC). The probability density function (PDF) for PKMC was designed using the binomial theorem as described below. The binomial theorem facilitates the application of dynamic choice of higher weights to small cluster(s). The algebra related to the binomial theorem is relegated in Additional file 1. In the case study considered here, ideally one would see three clusters to represent the background, RBC and stained pixels. The corresponding probability density function (PDF) generated for these 3 clusters is shown in Figure 12.

**Figure 13:**
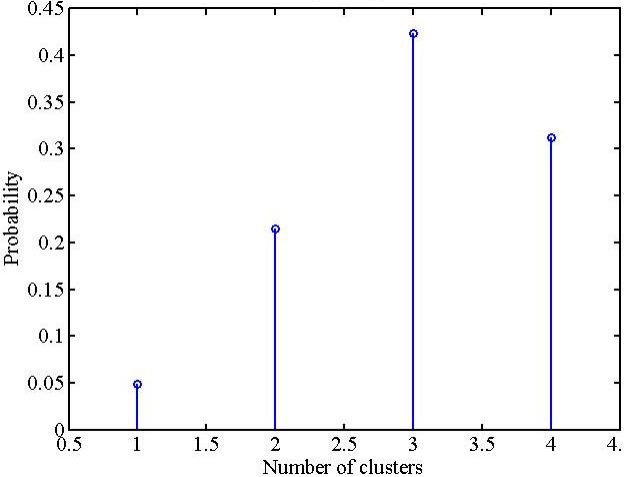
**Probability density function for 4 clusters**.

In reality some artefacts (such as the central pallor) could also show up as a separate cluster. In this case, the design parameters will be: the number of clusters (4 in this case), and the stained pixels would be represented by the third cluster. The resulting probability density function (PDF) from the Binomial theorem maximisation is shown in Figure 13.

The discrete probability density function designed as described (in Additional file 1) was used to select weights for the clusters. The stained pixels were given higher weights and lower weights were assigned to other clusters so as to retain parasite information buried in a large population.

The PKMC labels the clusters as follows: first cluster for the background, the second cluster for the stained pixels and the third cluster for the RBCs. The PKMC also gives the average intensity of each cluster and designates this as the cluster centroids. The last part of the algorithm is concerned about how to separate artefacts from the potential parasites. The artefacts can be visualized as stained WBC, platelets, dirt or dye crystals. To still enhance the sensitivity of the method PKMC work is in progress to develop clustering over true RBG (Red, Blue and Green channel) images instead of gray-scale images.

### Potential parasite confirmation

**Figure 14:**
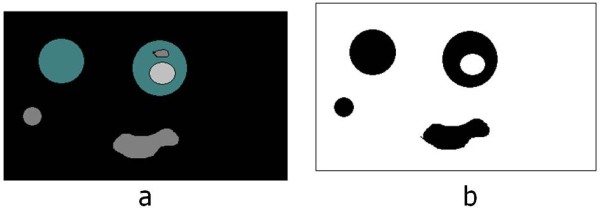
**a) Gray scale image with background pixels set to zero (step 2) b) Binary image after boundary detection (step 5)**.

**Figure 15:**
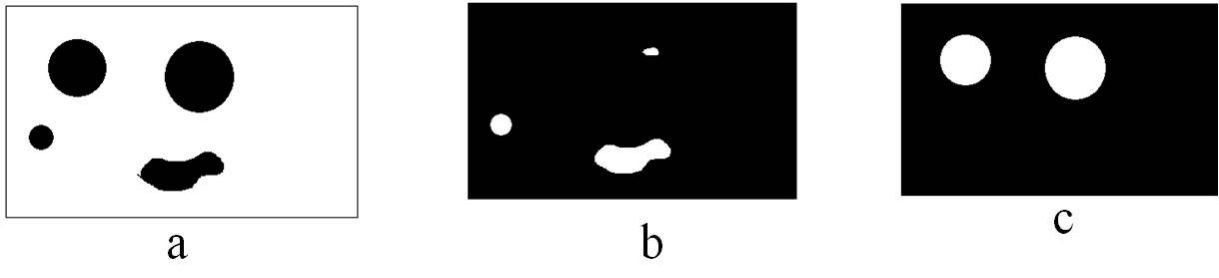
**Ideally PkMC generates three clusters a) Background, b) Stained pixels (parasites, platelets and other artefacts) and c) RBC**.

**Figure 16:**
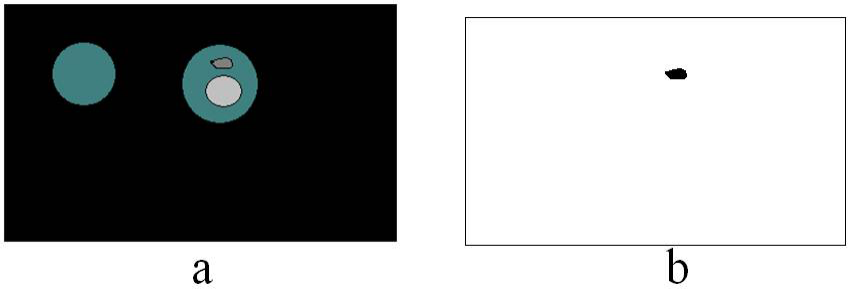
**After implementing threshold check a) Only RBCs are retained i.e. platelet and artefact are removed to finally yield b) Binary mask of Parasite**.

The features of parasite need to be understood to separate them from artefacts. An intelligent separation scheme can be designed by exploiting the features such as: parasites are embedded with in the RBC and artefacts are more or less homogeneous objects. The method to separate parasites from the artefacts is described below by an illustrative example. Figure 14a represents a sample image after background intensity set to zero and with a parasite in one of the RBCs. Figure 14b is binary image of Figure 14a. Now each component in Figure 14a will have an average intensity and clearly the average intensity of platelet or WBC will be near to stained pixel intensity because of their homogeneous nature. An intensity based threshold can be used to separate the platelet and WBCs. Recall that PKMC returns three clusters of a) background, b) stained pixels and c) RBC as shown in Figure 15. Apart from clusters labels PkMC also reports the average intensity of each cluster as three centroids. The comparison of the average intensity of each component from Figure 14a to the centroid for stained pixel helps to distinguish between platelet or WBCs or any other artefact. This ensures that stained pixels, which are parasites, are retained and artefacts are removed as shown in Figure 16.

**Figure 17:**
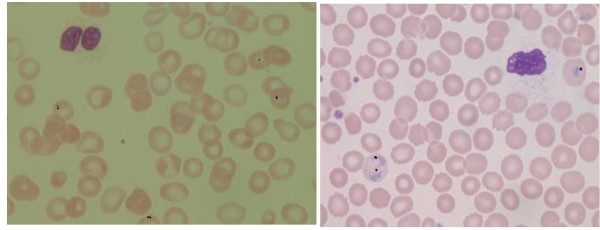
**Parasites marked image**.

The parasite detection method was implemented over the 10 potential positive cases and 10 negative cases. The identity of the positive and negative cases was unknown at the time of examination. The detection of parasites in two examples is represented in Figures 17a and 17b are marked in black. The importance of the unsupervised algorithm has been validated on images taken in different condition. Also the platelets and WBCs in such images are not recognized as parasites.

## Results

The proposed method was tested on large variety of images with malarial parasites at different life stages to check for the sensitivity and specificity of the algorithm. The method is developed using few images from Alberta Health Services and then tested over different variety cases and individual images. The algorithm is designed to work on stack of images and provide cumulative results or it can generate results on a single image. The performance of the method was tested on cases provided by Alberta Health Services laboratories for 10 potential positive cases of malaria and 10 negative cases to cross-validate the method. The method was further verified and tested in a blindfold format on over 40 images from a data set provided by the Department of Pathology and Laboratory Medicine, at the University of Washington. These results clearly show the robustness and practicality of the proposed method. The measure of performance and accuracy of the method was evaluated by two metrics: sensitivity and specificity [9]. Sensitivity is defined as the probability (percentage) that patients with the infection will have a positive result using the test under evaluation. Specificity is defined as the probability (percentage) that patients without the infection will have a negative result using the test under evaluation. The values for sensitivity and specificity are expressed in terms of true positives (TP), false positive (FP), false negative (FN) and true negative (TN) as defined below in expressions 2 and 3:

(2) $$Sensitivity=\frac{TP}{TP+FN}$$

(3) $$\begin{array}{c} Specificity=\frac{TN}{TN+FP} \end{array}$$

The results of all the cases are summarized below, where the values of false positives, false negatives, true positives and true negatives are reported in Tables 2 and 3. Table 2 is prepared using blood slide images provided by Alberta Health Services in which there are 20 cases in total, 10 of which are positive (parasites present) and 10 are negative (no parasites) cases and each case consists of 10 images. Whereas Table 3 is prepared using blood slide images provided by Department of Pathology and Laboratory Medicine, University of Washington, the 41 randomly images chosen for the analysis contain a large variety of images of which 12 had parasites while 29 contained no parasites and these results are summarized in Table 3.

**Table 2:** Results for detecting and confirming parasites from blood slide images provided by Alberta Health Services

Total cases	20
True positives	10

False positives	5

False negatives	0

True negatives	5

Sensitivity %	100

Specificity %	50%

**Table 3:** Results for detecting and confirming parasites from blood slide images provided by the Clinical Microbiology Laboratory, Harborview Medical Center, Seattle, WA

Total cases	41
True positives	37

False positives	4

False negatives	0

True negatives	29

Sensitivity %	100%

Specificity %	88%

Tables 2 and 3 give a good insight into the efficacy of the automated diagnosis process and the decision making by the algorithm. The large number of false positives can be attributed to the presence of higher number of artefacts and poor image quality. In any case the algorithm serves as a screening tool and is designed to be overly sensitive so that no true cases of malarial parasitaemia are missed. The benefit of the unsupervised method proposed here is readily evident from the minimal or no cases of false negatives.

Apart, from the combined results of all the cases, a detailed study for one random case is also presented. The results from each of the 10 images for this random case are reported in Table 4. This table gives a comparative study between results from the proposed algorithm and results from the pathologist. This study shows the sensitivity and accuracy of the proposed method. The errors in the iRBC count were due to high platelet count.

**Table 4:** Results for detecting patient M7, reported parasitaemia = 2

	Algorithm result		Pathologist's result	
	**RBC count**	**iRBCcount**	**RBC count**	**iRBCcount**

M7-1	67	0	66	0

M7-2	74	2	74	2

M7-3	64	3	64	3

M7-4	75	0	73	0

M7-5	64	1	64	1

M7-6	76	2	76	1

M7-7	75	3	75	3

M7-8	72	5	71	1

M7-9	64	1	66	1

M7-10	74	1	75	1

## Discussion

The parasitaemia level is a crucial factor to report and depends on the sections or fields of image observed. This is a fundamental limitation of manual microscopy, that is the ability to detect malaria depends on the number of microscopic fields observed [16]. The automated technique presented here is successful in detecting a single parasite in a microscopic field.

The advantage of the method presented in this paper is that, even in the case of examination of an entire slide, it is able to significantly reduce the effective time of diagnosis when combined with an image acquisition system. Secondly, the unsupervised nature of the method will minimize human reliance and enforce consistency in reporting the level of parasitaemia and will bring down the effective cost of diagnosis. Lastly, the method not only focuses on detecting parasites, it also reports the size distribution of RBCs which could lead to the diagnosis of other diseases related to the size or abnormal size distribution of RBCs. This paper provides an automated, consistent, robust and unsupervised screening method for malarial parasites. Existing diagnostic methods in the literature depend extensively on skilled operators and require training. Compared to other diagnostic techniques, the reported method avoids problems associated with "rapid diagnostic" methods, such as being species specific and high per test cost. In contrast the proposed method is completely automated, once slides are made and imaged. The method was specifically developed to work on low resolution images and thereby removes dependency on high resolution microscopy which boosts the practicality of the algorithm. Combined with an image acquisition system this method will allow entire slide to be examined, minimizing human reliance. The necessity of reliable electricity is one principal obstacle in using this kind of system in developing countries or outside of healthcare facilities. However it is anticipated that such an algorithm can easily be incorporated in a battery operated digital camera that would have as its output: 'negative' or 'positive with count of parasite(s)'. There are various vendors which provide camera enabled microscopes however suitable "hand held" image capture technology has yet to be developed for a tropical laboratory, cell-phone based technologies could also be contemplated.

In summary, we present an unsupervised screening method for thin smear analysis, which has the potential to be integrated into a diagnostic platform. In reference to development of artemisinin resistance the need for speedy and cheap screening and accurate iRBC count is presented. The method presented has potential application in generating parasite clearance curves which is an important measure of anti-malarial drug efficacy [17]. For this method to have clinical utility, close to 1,000 high power fields would need to be analysed- this could happen on a traditional glass slide or on a yet-to-be-developed substrate.

## Competing interests

The authors declare that they have no competing interests.

## Authors' contributions

YP carried out the image analysis work for this study as part of his MSc thesis under the supervision of the second author (SLS). Both YP and SLS were involved in the development of the image-based screening method and in the preparation the first version of this manuscript. AM, GC, and AA gave support and expertise as pathologists and provided images for analysis and validation of the proposed algorithm. They also advised on the needs and requirements of an unsupervised algorithm and provided many comments on the earlier and this final version of the manuscript. All authors have read and approved the final manuscript.

## Supplementary Material

Additional file 1 **Use of the binomial theorem for automated clustering**. Click here for file
