# Supplementary material for: Automated and unsupervised detection of malarial parasites in microscopic images
Source: Malar J. 2011 Dec 13;10:364. doi: 10.1186/1475-2875-10-364 (PMC3254597; doi:10.1186/1475-2875-10-364)
Supplement: Additional file 1 — Use of the binomial theorem for automated clustering. [file 1475-2875-10-364-S1.DOC]

**Additional file 1**

**Use of the binomial theorem for automated clustering**

The mathematical expression for the binomial theorem can be expressed as:

(x+y)^n^ = ^n^C_0_x^n^ + ^n^C_1_x^n-1^y + ... + ^n^C_k_x^k^y^n-k^ +… + ^n^C_p_x^p^y^n-p^ + ^n^C_n-1_xy^n-1^ + ^n^C_n_y^n^ (1)

Where:

$x,y \in R^{+}, n\in N$ and

^n^C_k_ = n!/k!(n-k)! (2)

The expression can be explained as follows: in total there are a total of *‘n’* clusters and the *k^th^* and *p^th^* clusters have to have the maximum weight. The case study considered in this research work has only one cluster with the maximum weight, so we can choose ‘*y’* as unity, leaving the variable *‘x’* free for maximization. The discrete probability distributions for different cases are described below with the maximization expressions.

**Case 1:** When *‘n’* is even and for a given *k^th^* cluster;

p(k) = ^n^C_k_ x^n-k^/{(1+x) ^n^ - x^n^} (3)

Equation 3 shows that the probability of *k^th^* cluster depends on *x, k* and *n*. Mathematically, it can be shown that *p(k)* will have a global maximum value for a particular value of *x* for a given *k* and *n*. The advantage of using this form of probability function with given *‘n’* and *‘k’* is that we can solve for *‘x’*, such that *p(k)* is maximum.

The value of *‘x’* for maximising *p(k)* can be solved by setting the derivative of *p(k)* with respect to *‘x’* to zero. The equation derived after equating the derivative to zero can easily solved for a positive root of *‘x’* under the constraint that *0 < p(k) < 1:*

n(1+x) ^n-1^ – k(1+x)^n^ + kx ^n^ = 0 (4)

**Case 2:** When *‘n’* is odd for a given *k^th^* cluster;

*Sub case 1:* *1 ≤ k ≤ (n-1)/2*

p(k) = ^n^C_k_ x^n-k+1^/{(1+x) ^n^ - ^n^C_(n-1)/2_ x^(n+1)/2^} (5)

Similar to the maximization scheme described earlier, the value of *‘x’* can be solved by setting the derivative of *p(k)* with respect to *‘x’* to zero. The equation derived after equating the derivative to zero can be solved for a positive value of *‘x’* under the constraint *0 < p(k) < 1*:

n(1+x) ^n-1^ +(1-k)(1+x)^n^ + {(2k-n-1) ^n^C_(n-1)/2_ x^(n+1)/2^}/2 = 0 (6)

*Sub case 2: (n-1)/2< k ≤ n*

p(k) = ^n^C_k_ x^n-k^/{(1+x) ^n^ - ^n^C_(n-1)/2_ x^(n+1)/2^} (7)

n(1+x) ^n-1^ - k(1+x)^n^ + {(2k-n+1) ^n^C_(n-1)/2_ x^(n+1)/2^}/2 = 0 (8)
